# EMG Biofeedback for online predictive control of grasping force in a myoelectric prosthesis

**DOI:** 10.1186/s12984-015-0047-z

**Published:** 2015-06-19

**Authors:** Strahinja Dosen, Marko Markovic, Kelef Somer, Bernhard Graimann, Dario Farina

**Affiliations:** Department of Neurorehabilitation Engineering, University Medical Center Göttingen (UMG), Georg-August University, 37075 Göttingen, Germany; Otto Bock HealthCare GmbH, 37115 Duderstadt, Germany

**Keywords:** Closed-loop prosthesis control, Myoelectric prosthesis, EMG biofeedback, Sensory feedback, Routine grasping, Force steering, Grasping consistency

## Abstract

**Background:**

Active hand prostheses controlled using electromyography (EMG) signals have been used for decades to restore the grasping function, lost after an amputation. Although myocontrol is a simple and intuitive interface, it is also imprecise due to the stochastic nature of the EMG recorded using surface electrodes. Furthermore, the sensory feedback from the prosthesis to the user is still missing. In this study, we present a novel concept to close the loop in myoelectric prostheses. In addition to conveying the grasping force (system output), we provided to the user the online information about the system input (EMG biofeedback).

**Methods:**

As a proof-of-concept, the EMG biofeedback was transmitted in the current study using a visual interface (ideal condition). Ten able-bodied subjects and two amputees controlled a state-of-the-art myoelectric prosthesis in routine grasping and force steering tasks using EMG and force feedback (novel approach) and force feedback only (classic approach). The outcome measures were the variability of the generated forces and absolute deviation from the target levels in the routine grasping task, and the root mean square tracking error and the number of sudden drops in the force steering task.

**Results:**

During the routine grasping, the novel method when used by able-bodied subjects decreased twofold the force dispersion as well as absolute deviations from the target force levels, and also resulted in a more accurate and stable tracking of the reference force profiles during the force steering. Furthermore, the force variability during routine grasping did not increase for the higher target forces with EMG biofeedback. The trend was similar in the two amputees.

**Conclusions:**

The study demonstrated that the subjects, including the two experienced users of a myoelectric prosthesis, were able to exploit the online EMG biofeedback to observe and modulate the myoelectric signals, generating thereby more consistent commands. This allowed them to control the force predictively (routine grasping) and with a finer resolution (force steering). The future step will be to implement this promising and simple approach using an electrotactile interface. A prosthesis with a reliable response, following faithfully user intentions, would improve the utility during daily-life use and also facilitate the embodiment of the assistive system.

**Electronic supplementary material:**

The online version of this article (doi:10.1186/s12984-015-0047-z) contains supplementary material, which is available to authorized users.

## Background

Human hand is a dexterous end-effector and a sophisticated instrument for sensory exploration [1]. After an amputation, these important motor and sensory functions are abruptly lost. Myoelectric hand prostheses can be used to restore grasping. The control signal (input voltage) driving the prosthesis motor is obtained by applying simple processing (smoothing) to the electromyography (EMG) signals recorded from the user muscles. The commercial state-of-the-art myoelectric interface uses two channels of EMG: the activity of hand and wrist flexor muscles is proportional to the prosthesis closing speed and grasping force, while the extensor activity controls proportionally the speed of opening [2, 3]. Therefore, the commercial myoelectric prostheses provide the grasping function by restoring the feedforward pathway between the user’s brain and the artificial hand, but there is no sensory feedback from the prosthesis to the user. There is only one commercially available system [4], presented recently, implementing a simple feedback about the hand grasping force. In principle, sensor data can be transmitted from the prosthesis to the user invasively, through a direct stimulation of the nerves [5], and non-invasively, by electrically [6] and/or mechanically [7] stimulating the skin. Closing the loop in myoelectric prostheses was acknowledged as an important future goal by the prospective users as well as researchers in the field [8]. Sensory feedback might improve the utility of the assistive devices as well as facilitate the embodiment [9].

Two-channel myoelectric interface is a simple and intuitive control method since the user operates the prosthesis by activating the same muscles (finger flexors/extensors) that were responsible for those functions (hand open/close) before the amputation. However, the EMG signals acquired using surface electrodes are noisy and variable, due to inherent limitations of the recording setup (e.g., detection separated from the signal source), and the control is thereby rather imprecise [10]. For this reason, as demonstrated in [11], the prosthesis may respond inconsistently to the user intentions. Repeatedly closing the prosthesis to generate the same grasping force was characterized with a large variability, which also increased with higher target forces. The subjects could not repeat muscle contractions in a reliable manner using the natural proprioceptive feedback from own muscles to provide consistent control signals. Imprecise control can produce user frustration, often leading to the abandonment of the prosthesis [12]. Furthermore, it can be a limiting factor for the effectiveness of the sensory feedback [11]. Indeed, it can be rather useless for the user to sense the state of the system (e.g., aperture or grasping force), if he/she cannot produce a sequence of commands driving the prosthesis reliably towards the desired state (e.g., target aperture or grasping force). Improving the consistency of the command is thereby an extremely relevant goal. A reliable control loop would allow the benefits of the sensory feedback to be fully expressed. A well-controllable prosthesis following faithfully the user intentions would also better emulate the operation of its biological counterpart, potentially facilitating embodiment.

In the current study, we propose a novel concept for closing the loop in myoelectric prostheses, designed specifically to improve the consistency of the prosthesis response by allowing the user to reduce the variability of the control signals he/she generates by muscle activation. The new approach was tested experimentally and the tests demonstrated that it significantly improved the performance both in routine grasping and force steering tasks.

## Methods

### Proposed method

#### General concept

**Fig. 1:**
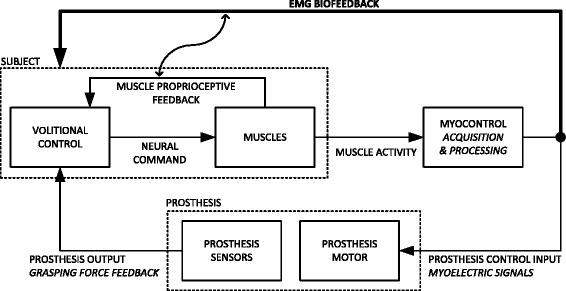
Conceptual scheme for the application of EMG biofeedback to improve force control in myoelectric prostheses. The user receives online information about the level of the myoelectric signals he/she generates. Since the grasping force is approximately proportional to the input myoelectric signals at the moment of contact, the user can control the grasping force predictively, i.e., by adjusting his/her myoelectric signals during the prosthesis closing. EMG biofeedback can also facilitate the modulation of the grasping force once the hand is closed (see text)

In the classic approach to closing the loop in myoelectric prostheses, the system output (e.g., grasping force) is delivered to the user. The novel concept proposed and investigated in this study (Fig. 1) is to provide feedback on the control input that the user generates (prosthesis command) in addition to the consequence of such input (grasping force). Specifically, the generated and processed myoelectric signals are transmitted to the prosthesis as commands and simultaneously to the user as online feedback information. In the conventional approach to prosthesis control, the myoelectric signals are latent variables, whereas in the novel scheme (Fig. 1) these signals become explicit (observable) through the application of the EMG biofeedback. The user can therefore modulate the control input by using a local closed loop (bold line in Fig. 1), allowing him/her to produce consistent and reproducible commands, actively compensating for the inherent variability of the surface myoelectric interface. The proposed method was tested in two representative prosthesis control tasks, namely, routine grasping and force steering.

#### Routine grasping task

Routine grasping refers to a smooth and straightforward closing of the prosthesis so that the desired grasping force is reached immediately after contacting the object, avoiding thereby a careful (and tedious) adjustment of the prosthesis force [11]. This resembles the way in which able-bodied persons grasp objects in daily life. When using a prosthesis, the routine grasping is accomplished by generating and holding a certain level of muscle contraction (as a percent of the maximum voluntary contraction, MVC); since the closing speed and grasping force are proportional to the command input, the prosthesis closes at a certain speed (as a percent of the maximum speed), which becomes “converted” into a corresponding force (percent of the maximum force) once the motor stalls (contact with the object). In a conventional closed-loop system, the user regulates his/her myoelectric output indirectly, by modulating the intensity of contraction relying solely on the proprioceptive feedback from own muscles. The user is therefore unaware of the exact control signal that is being delivered to the prosthesis. Only after contact, the user receives the force feedback, which also reveals the actual command that was applied to the prosthesis during closing; however, this information comes too late since the grasp is already formed (e.g., object broken due to an excessive force). When the EMG biofeedback is provided, as proposed in the novel scheme (Fig. 1), the task becomes explicit. The user is able to modulate the muscle activity reaching the desired signal level (as a percent of MVC) and then maintain that level by relying on the EMG biofeedback closed loop. The hand starts closing, and the user, by monitoring and controlling his/her myoelectric activity, predictively controls the level of force that will be generated once the object is contacted (grasped).

#### Force steering task

In this task, the aim is to modulate the grasping force while the hand is closed around an object (e.g., grasping an object and then strengthening the grip) [13]. There are two mechanisms characteristic for myoelectric prostheses making the modulation of force challenging.

First, the prosthesis is non-backdrivable, allowing the user to relax the muscles while the prosthesis continues holding the attained level of force. This frees the user from having to maintain a prolonged muscle contraction. When the force needs to be increased, however, the user must activate the flexor muscle and increase the contraction from the resting state until the control signal is higher than the level corresponding to the current grasping force. Since in the classic control scheme, the user does not know the exact value of the control signal that is being generated, he/she cannot be sure when the prosthesis will start reacting. Therefore, the eventual increase in force often comes as a surprise, leading to a poor control of the force increments. By providing the EMG biofeedback, the moment the prosthesis will respond becomes explicit, since the user can monitor online (and precisely modulate) how the control input approaches the current level of force.

Second, a completely different mechanism is active when decreasing the force. In this case, the user releases the grip by commanding the prosthesis to open, where the velocity of opening is proportional to the myoelectric signal recorded from the extensor muscle. In order to decrease the force gradually, the hand must be opened very slowly, by activating the extensor just above the threshold level. Again, in the classic approach, this is difficult to accomplish since the current level of the generated myoelectric signal is unknown to the user. Often, the prosthesis force suddenly drops to zero as a result of a higher extensor activation, which opens the hand and breaks the contact with the object. Again, with the EMG biofeedback, the user can fine-tune the low-level control signal and thereby decrease the force gradually and in a controllable manner.

In both of these tasks (force steering and routine grasping), the EMG biofeedback can be regarded as assisting and enhancing already existing natural proprioceptive feedback from the muscles, which alone is not a reliable indication of the level of muscle contraction and prosthesis response [11]. In that sense, the EMG biofeedback can be applied as a training instrument facilitating the subject to better utilize (interpret) the natural muscle proprioceptive feedback for prosthesis control. This is denoted in Fig. 1 by the “S”-shaped arrow connecting the two feedback channels. However, this potential application of the EMG biofeedback was outside the scope of the current study.

### Experimental setup and protocol

**Fig. 2:**
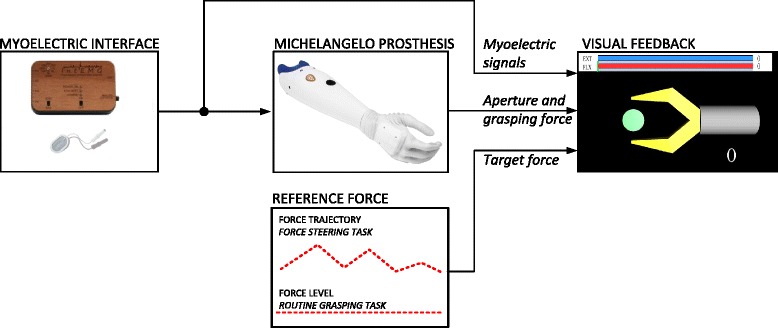
The components comprising the experimental setup. A myoelectric interface was used to proportionally control the Michelangelo hand prosthesis using hand and wrist flexor and extensor muscles. The subject was visually- and sound-isolated from the real prosthesis and instead monitored the virtual gripper shown on the computer screen. The gripper received online sensor data from the prosthesis (aperture and force) and thereby replicated the behavior of the real system. The virtual scene also included the visual feedback about the generated force, target force and myoelectric activity from the flexor and extensor muscles

The setup comprised: 1) Michelangelo Hand prosthesis (Otto Bock Healthcare Products GmbH, Vienna, AT), 2) EMG amplifier (INTEMG, OTBioelettronica, IT), and 3) a standard desktop computer with a 22” screen. Figure 2 depicts the components and the control loop as it was implemented in the real-time framework for the assessment of the manual closed-loop control systems [14].

**Fig. 3:**
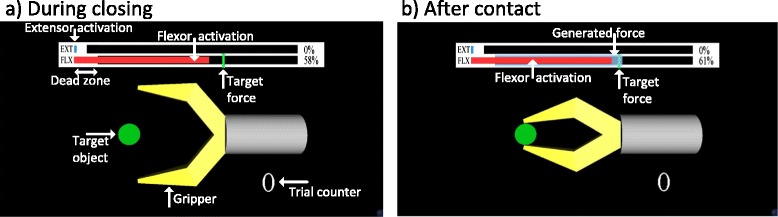
Visual scene shown to the subjects during the experiments including a snapshot of the screen (**a**) before contact and (**b**) after contact. The real prosthesis (Michelangelo hand) hidden from the subjects’ view grasped a stiff cylindrical object, and this was presented to the subject in the form of a virtual gripper grasping a virtual target object. Horizontal bars, red for the flexor and blue for the extensor, showed a continuous feedback about the current level of muscle activity (prosthesis control signals). As long as the myoelectric bars did not reach the respective black lines, the myoelectric activity was subthreshold (i.e., a dead zone area resulting in zero control input to the prosthesis). Semi-transparent blue bar indicated the hand grasping force and the green vertical line was the target force level. During the routine grasping, the target force was stationary, while in the force steering task, it was moving according to the time profile of a reference force trajectory

The Michelangelo Hand [15] is a two degree-of-freedom prosthesis with mechanically-coupled fingers flexing and extending around the metacarpophalangeal joints plus the thumb which can also move into opposition. Therefore, the hand can implement lateral (between the thumb and index) and pinch (between fingertips) grasps, where only the latter was used in the current study. The hand integrates a Bluetooth interface through which a normalized command signal can be sent to the prosthesis. The hand response profiles mapping the constant command input to the closing speed and grasping force, respectively, were recorded and then linearized to obtain an ideal correspondence (i.e., X% of MVC ⇨ X% of maximum speed ⇨ X% of maximum force). Two channels of bipolar EMG were recorded from the hand and wrist flexor and extensor muscles, proportionally controlling the hand closing/opening and grasping force. Standard pre-gelled Ag/AgCl electrodes were used (Neuroline 720, Ambu, US). A stiff cylindrical object was positioned and secured between the prosthesis fingers so that the hand grasped it when closed. During the experiment, the prosthesis and the object were placed in another room, while the subjects were looking into the computer screen showing a geometrical model of a simple gripper grasping a stiff cylinder (Fig. 3). Therefore, the subjects controlled the real prosthesis (Michelangelo Hand) through the myoelectric interface. The prosthesis sensor data (position and force) were sampled internally by the embedded controller (100 Hz) and then sent to the host PC to update the visual feedback displayed on the computer screen. The gripper replicated the movement (aperture) of the prosthesis and the grasping force was displayed using a horizontal bar, as described below. The setup provided a standardized feedback across subjects and conditions. By detaching the subjects from the prosthesis, some sources of feedback were eliminated (e.g., motor/mechanism sound, haptic feedback through the socket, deformation of the silicone skin when grasping an object). However, as in a real-life application, the subjects could still monitor the prosthesis movements, and the setup was configured specifically to facilitate this observation (e.g., clear, lateral view of the prosthesis). This was done considering that the prosthesis closing velocity is an important information, since it can be used to control the grasping force predictively, as demonstrated in [11].

The EMG was sampled at 1 kHz and its root mean square was computed over time intervals of 250 ms and with 90 % of overlap. The control loop running at the PC (Fig. 1) operated at 200 Hz. The acquired data from the EMG amplifier were sent to the PC via USB, filtered using a first-order low-pass Butterworth filter with a cutoff at 1 Hz, and finally thresholded and normalized to the interval [0, 1], where 0 and 1 corresponded to the sub-threshold activity and 70 % of MVC, respectively. This was done in order to map the prosthesis force range to the user sense of effort (high force, high effort) but still avoid fatigue during repeated contractions. The exact level used in the present experiment was adopted based on pilot tests, since to our knowledge there are no studies investigating the optimal mapping between the prosthesis force and user myoelectric range. In practice, amputees adjust this mapping according to personal preferences by, for example, turning a potentiometer on the electrode (Otto Bock systems). The resulting command signal was sent to the hand prosthesis and to the block implementing the virtual scene on the computer screen. The scene (Fig. 3) included a geometrical model of a simple gripper and horizontal bars just above the gripper, providing the current value of the flexor and extensor myoelectric signals (EMG biofeedback) as well as the generated and target grasping force (classic force feedback). Note that during the prosthesis closing (Fig. 3[a]), while the grasping force was zero, the subject activated his/her flexor muscle so that the generated myoelectric signal (red bar) was close to the target force level (green line). As a result, after contact (Fig. 3[b]), the generated grasping force (semi-transparent blue bar) reached close to the desired level. Once the grasp was formed, the prosthesis reacted by increasing the force only when the myoelectric activity was higher than the current force level (i.e., red bar overtaking the semi-transparent blue bar); otherwise, the prosthesis held the current force due to the non-backdrivable operation. Similarly, the prosthesis started decreasing force only after the extensor signal crossed the dead-zone threshold (i.e., blue bar crossing into the respective black line). As explained before, the aim of the current study was to present and for the first time test the validity and benefit of the novel approach. Therefore, an ideal visual feedback was used to transmit the information to the user. However, the information transmission can be easily translated into another modality, e.g., electro- or vibrotactile, as discussed later.

Ten able-bodied subjects (23 ± 3 years) and two amputees (55 and 43 years) participated in the study, and signed the informed consent forms for the experiment approved by the Ethical Committee of the University Medical Center Göttingen. First amputee, hereafter denoted as amputee 1, was an experienced and active user (50 h/week) of a myoelectric prosthesis (Sensor Hand, Otto Bock), with the left hand amputated 30 years ago at the transradial level. Second amputee (amputee 2) was congenital (wrist level, right hand), and also experienced but occasional user (10 h/week) of the same type of myoelectric prosthesis. The subjects were comfortably seated in a chair in front of a table, looking into a computer screen positioned approximately 50 cm away. The positions for the placement of the EMG electrodes were determined by palpating and visually observing muscle contractions in the dominant forearm of able-bodied subjects and residual limb of amputees, and the skin was prepared with a small amount of abrasive gel (everi, Spes Medica, IT). The forearm and hand of able-bodied subjects were placed within an orthopedic splint so that the subjects controlled the prosthesis by generating nearly isometric muscle contractions. The arm was held in a self-selected comfortable position (e.g., vertically next to the trunk or on the table). The principle of prosthesis operation was explained to the subjects and they were allowed to practice both tasks for a short time (10–15 min).

The task for the subjects during the routine grasping test was to close the prosthetic hand from the fully open position, grasp the object and reach the desired level of force as indicated by the target force bar. The subject then relaxed the muscles to mark the end of the trial, and this triggered an automatic opening of the hand. The maximum force attained during the trial was adopted as the trial outcome. The subjects were instructed to activate the muscles and close the hand so that the target grasping force was reached directly after contact (no force steering). During training, if the experimenter noticed that the subjects corrected the force after contact, he discouraged them from doing so in the next trials. In addition, the control algorithm ignored any extensor input from the user (no force decrease). The subjects grasped repeatedly in two blocks of 50 trials with the target forces equal to 30, 50 and 70 % of the maximum, with simultaneous EMG and force feedback (EMG/FORCE, novel approach) and with force feedback only (FORCE, classic closed-loop scheme). In the latter condition, the bars indicating the current level of muscle activity (Fig. 3, red for flexor, blue for extensor) were not shown. At the beginning of the trial, the target force (vertical green line) was displayed, and after contact, the momentary grasping force (semi-transparent blue bar) was indicated to the subject. In total, there were 300 trials in both feedback conditions. The first ten trials in each block were regarded as a warming up and were not used for data analysis. Due to a routine grasping paradigm, the trials were fast and lasted few seconds; to reach the target force, the prosthesis had to be closed at a certain velocity and this determined the trial duration. For example, for 70 % target force, the time from the start of the prosthesis closing to reaching a stable grasping force was less than 2 s.

In the force steering test, the task was to control the force of an already closed prosthesis so that it tracked a 110-s long pseudorandom reference trajectory comprising a sequence of gradual, increasing and decreasing slopes. This time the subjects had to control manually both force increase and decrease using flexor and extensor muscles, respectively (no auto-open). The reference force level was indicated by the target force bar (Fig. 3, green line) moving according to the time profile of the reference trajectory, and the task for the subject was to produce the muscle activity generating the grasping force that would track the moving reference as close as possible (Fig. 3, semi-transparent blue bar following the green target line). The subjects performed four tracking trials using simultaneous EMG and force feedback (EMG/FORCE, novel approach) and force feedback only (FORCE, classic approach). The first trial was regarded as a warming up and was not used for the data analysis. In both routine grasping and force tracking, the order of the feedback conditions was randomized between the subjects.

### Data analysis

The variability of the generated forces was expressed as interquartile range (IQR) and used to evaluate the consistency in the control of force (i.e., precision). The accuracy was assessed by computing the absolute error defined as the absolute value of the difference between the generated and desired grasping force. Bartlett multiple-sample test for equal variances was applied to determine statistically significant difference in dispersions within the conditions overall, followed by Ansari-Bradley two-sample test with Bonferroni correction for pairwise comparisons of the force variability between the conditions. The quality of force tracking was assessed by calculating the root mean square tracking error (RMSE) between the generated and reference force profiles. All the results were reported as normalized forces, either in fractions or percent, i.e., 1 or 100 % corresponded to the maximum force of the prosthesis (~100 N). The stability of force control during force steering was assessed by determining the number of sudden drops in force. A drop was detected if the force fell below 10 % over those segments of the reference trajectory where the reference force was 20 % and higher. The statistically significant difference in absolute errors during the routine grasping and in RMSE during the force tracking between the two feedback conditions were evaluated using Wilcoxon signed rank test, as the data did not pass the normality test (one sample Kolmogorov-Smirnov). The threshold for the statistical significance was adopted at *p* < 0.05.

## Results

### Routine grasping

**Fig. 4:**
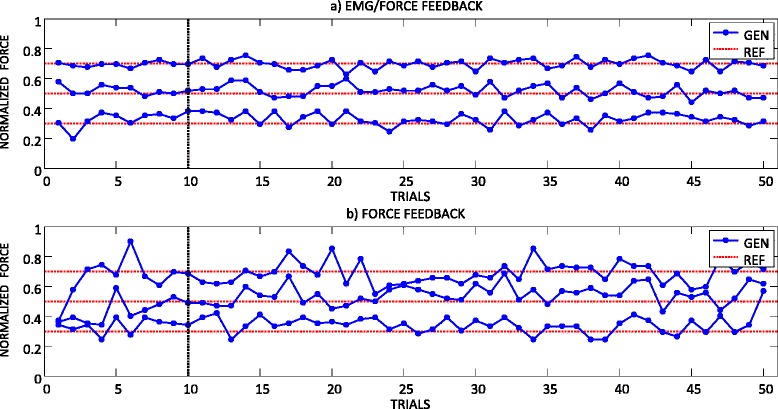
A representative result from an able-bodied subject performing routine grasping with (**a**) EMG and force feedback, and (**b**) force feedback only. The blue dots are the generated forces (GEN) and the red dashed lines are the target force levels (REF). The vertical black lines denote the 10^th^ trial

Figure 4 shows a representative result from an able-bodied subject performing the routine grasping task in two feedback conditions (EMG/FORCE and FORCE) and with three levels of target force (30 %, 50 % and 70 %). When the EMG biofeedback was provided (Fig. 4[a]), the generated forces were stable and consistent across trials, i.e., the points closely concentrated around the corresponding reference force levels. The lines connecting the points were parallel and well separated. With the force feedback only (Fig. 4[b]), the generated forces were more variable across trials. The connecting lines deviated from the reference, sometimes closely approaching the (wrong) neighboring force level. In addition, the subjects spent few initial trials (<10) tuning the prosthesis control in order to reach the desired force. The initial contractions for 50 and 70 % target were too low and the subject gradually increased the strength, through several trials, before finally arriving into the vicinity of the desired force. When the EMG biofeedback was provided, there was no need for this iterative adjustment, i.e., the subjects used the feedback to adjust the muscle contraction, generating the myoelectric signal that was close to the reference, and thereby producing the desired level of force already in the first trial (zero warmup).

**Fig. 5:**
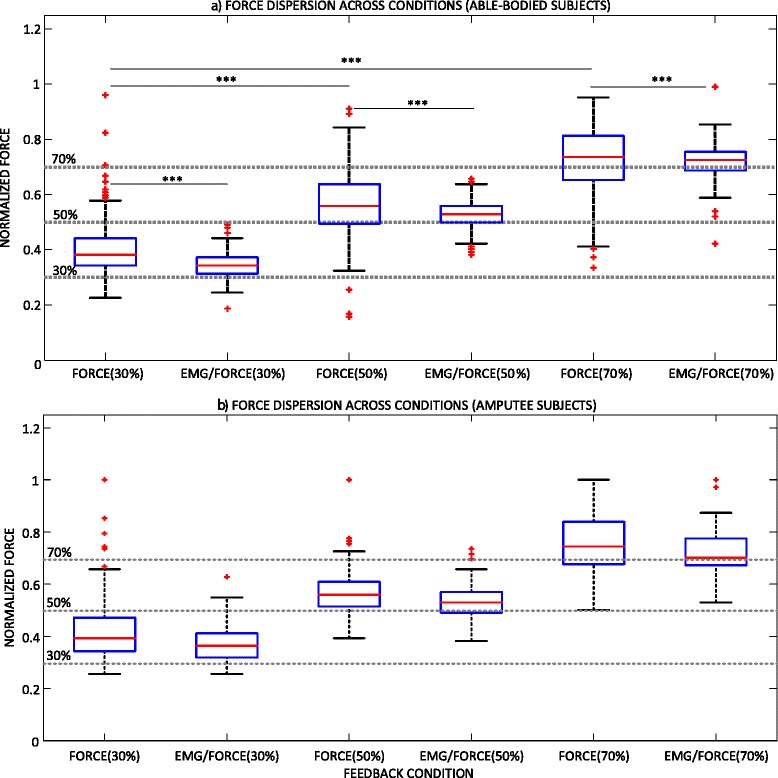
Summary results for the (**a**) able-bodied subjects and (**b**) amputees, performing the routine grasping task in two feedback conditions and at three target force levels. Boxplots depict the median (red line), interquartile range (blue box), maximal/minimal values (whiskers) and outliers (red crosses). Dashed gray lines are the target force levels. Horizontal continuous lines denote statistically significant differences in the force dispersions between the conditions (***, *p* < 0.001)

Summary results for the able-bodied subjects and all conditions are presented in Fig. 5(a). Providing the EMG biofeedback significantly improved the consistency in generating the grasping forces at all three force levels. Without the EMG biofeedback, the IQR was 10 %, 14 % and 16 % for the target force of 30 %, 50 % and 70 %, respectively, and it was approximately twofold lower when the EMG biofeedback was transmitted (i.e., 6 %, 6 % and 7 %, respectively). With the force feedback only, the force variability increased significantly for the higher target forces (FORCE (30 %) vs. FORCE (50 %) and FORCE (70 %) in Fig. 5[a]), which is a known trend [11]. When the EMG biofeedback was present, however, the dispersion was similar across all target force levels (no statistically significant differences). The occasional outliers in the generated forces, characteristic for the routine grasping using myocontrol [11], were less far from the median force when the EMG biofeedback was provided. Finally, the absolute errors (mean ± standard deviation) from the desired forces were twice smaller with the EMG biofeedback (5 ± 4 % vs. 10 ± 8 %), and this difference was statistically significant (*p* < 0.001).

The results for the two amputee subjects are shown in Fig. 5(b), demonstrating the similar trend as in able-bodied subjects. The provision of the EMG biofeedback reduced the IQR of the generated forces from 13 %, 9 % and 16 % for FORCE to 9 %, 8 %, and 10 % for EMG/FORCE for the target forces of 30 %, 50 %, and 70 %, respectively. The relative improvement was however less than in able-bodied subjects. Likewise, the amputee subjects were more accurate in generating the target forces with EMG biofeedback, which reduced the absolute errors (mean ± standard deviation) from 11 ± 10 % for FORCE to 6 ± 6 % for EMG/FORCE.

### Force steering

**Fig. 6:**
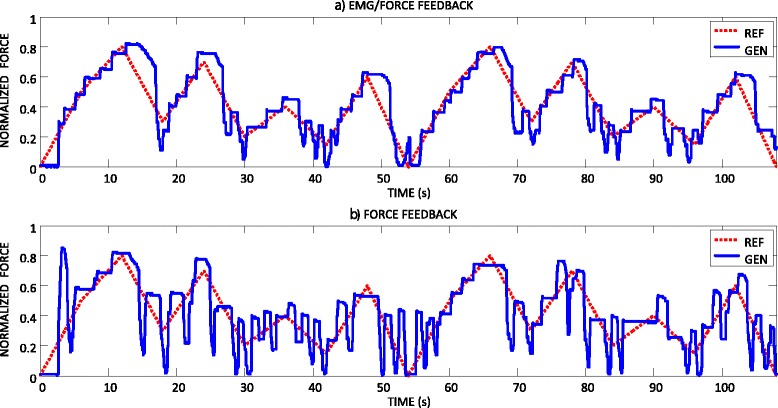
A representative result for the force tracking recorded from an able-bodied subject during (**a**) EMG and force feedback, and (**b**) force feedback only. The reference (REF, red dashed line) and generated (GEN, blue continuous line) force profiles are depicted

**Fig. 7:**
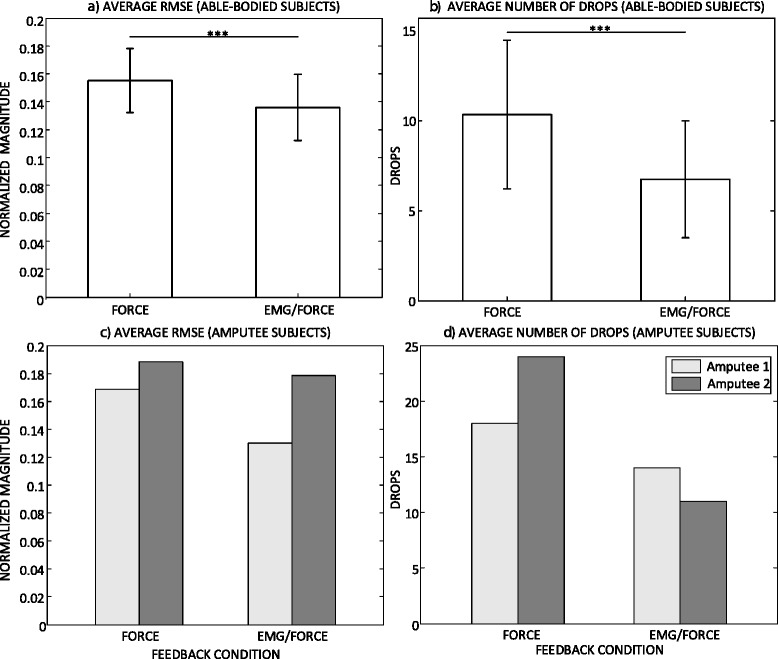
Summary results (mean ± standard deviation) for the force tracking task in two feedback conditions over all subjects: root mean square tracking error in (**a**) able-bodied and (**c**) amputees, and the number of force drops in (**b**) able-bodied and (**d**) amputees. The horizontal bar denotes statistically significant difference (***, *p* < 0.001)

The representative trials of force tracking recorded from an able-bodied subject in two feedback conditions are depicted in Fig. 6. In both cases, the generated force increased/decreased in sharp, discrete steps. This discontinuous modulation of force is an inherent characteristic of the prosthesis operation, related to e.g. intrinsic friction effects. However, with the EMG biofeedback, the steps were smaller in magnitude, and the generated force trajectory resembled the reference profile, although the resolution of the generated profile was coarser. With the force feedback only, the control of the force increment/decrement magnitudes was rather poor, and the generated trajectory oscillated around the reference with large under and overshoots. The overall profile of the reference was poorly represented in the generated trajectory. Several times, especially during the decreasing segments, the force dropped suddenly to zero. Summary results for the quality of tracking over all able-bodied subjects are given in Fig. 7(a) and (b). Providing the EMG biofeedback reduced the tracking errors. The decrease was modest but statistically significant (15.5 ± 2 % for FORCE vs. 13.5 ± 2 % for EMG/FORCE, *p* < 0.001). Similarly, the presence of EMG biofeedback improved the stability of tracking, since the number of force drops decreased from 10 ± 4 for FORCE to 7 ± 3 for EMG/FORCE (*p* < 0.001).

The results for the quality of tracking in amputee subjects are presented in Fig. 7(c) and (d). The outcome measures were better when EMG biofeedback was provided. The tracking errors decreased from 16.8 % and 18.8 % in FORCE to 13 % and 17.8 % in EMG/FORCE for the amputee 1 and 2, respectively. The number of drops in amputee subjects was higher compared to able-bodied, and it also decreased when using EMG biofeedback, from 18 and 24 in FORCE to 14 and 11 in EMG/FORCE for amputee 1 and 2, respectively.

## Discussion

A novel concept for closing the loop in myoelectric prostheses was demonstrated. In addition to feeding back the system output (generated grasping force), which is the classic method [9], in the novel approach the system input (myoelectric control signal) was also transmitted back to the user. The tests demonstrated that the provision of the EMG biofeedback improved the performance in both routine grasping and force tracking tasks. In the routine grasping, the online information about the prosthesis input allowed the subjects to adjust the motor command during the closing of the prosthesis so that the desired level of grasping force was achieved when the object was contacted. The subjects employed this simple predictive control scheme to anticipate the resulting grasping force. The EMG biofeedback also assisted the modulation of force while the prosthesis was closed (force tracking). During this task, the biofeedback allowed the subjects to monitor the ongoing myoelectric activity and compare it to the current thresholds for the prosthesis activation (force increase/decrease). With this, they were able to finely regulate the myoelectric signals around the respective threshold levels and thereby control the timing as well as the magnitude of the force increase/decrease, improving the effective resolution of the generated force trajectory as well as the stability of tracking (fewer force drops). The statistically significant but overall modest decrease in the RMSE reflects the inherent limitations of the force modulation mechanism in the prosthesis (force jumps) as well as the nature of the task (continuous force tracking). The reference force trajectory was such that the subjects gradually modulated the strength of the muscle contraction. The advantage of the EMG biofeedback might be even better expressed during a step force regulation: grasp an object with a certain force, relax muscles (prosthesis maintains the force), and then increase/decrease the force to a higher/lower force level. Importantly, the tests in two amputee subjects demonstrated that the EMG biofeedback can improve the performance even in experienced users of myoelectric prostheses. This is a preliminary but optimistic result that will be further evaluated in a future study including a larger pool of amputee subjects.

EMG biofeedback has been extensively used in the past in many fields of application, including rehabilitation, but the context was different [16]. For example, it is used during the user training to explain the principle of operation of the myoelectric prosthesis (e.g., as a didactic instrument) [17]. To our knowledge, this study is the first demonstration that the subjects can employ this type of information to improve the online control of the prosthesis grasping forces. The envisioned goal is to integrate this feedback as a standard component to enhance a daily-life prosthesis application. For the latter, the EMG biofeedback would have to be delivered through a tactile interface, as discussed later. Another possibility would be to implement the same protocol as in the current study by using a wearable augmented reality module (e.g., Google Glass). The module could connect to the prosthesis directly via a Bluetooth link and the EMG biofeedback bars could be shown on the wearable displays in the peripheral vision field. This was however outside the scope of the current proof-of-concept study. Nevertheless, even the current setup, with a host PC and the EMG biofeedback delivered on the computer screen, could be used as an instrument for the functional prosthesis training. It could assist the subjects in learning consistent force control, since it explicitly depicts the predictive mapping between the myoelectric command and the resulting grasping force. In addition, the EMG biofeedback could be utilized in daily life (electrotactile, augmented reality) or in the lab (host PC setup) to train the subjects to better exploit the natural proprioceptive feedback coming from their own muscles for the closed-loop prosthesis control. By controlling the prosthesis while assisted through the EMG biofeedback, the subjects could learn the mapping between the sensation of muscle contraction, including the sense of effort, and the resulting grasping force. After some time, this mapping could stabilize and even render the EMG biofeedback redundant. To investigate this possibility, a future study will include a multi-session biofeedback protocol. In that sense, it would be especially relevant to test this training in the subjects that are experienced in myoelectric control. These subjects might have already learned to utilize the muscle proprioceptive feedback for control and the EMG biofeedback might not improve the performance substantially. However, the preliminary tests in the present study as well as the results in [11] point out that this might not be the case.

The presented approach can be related to a model of the biological motor control [18, 19]. It is hypothesized that humans acquire internal models of the body dynamics and use them to control the movements in a predictive manner. By applying the motor commands to the forward models, the system can be simulated to predict the expected sensory consequences of the movement (reafference). The estimated reafference can then be used for the closed-loop control, compensating for the delays that are inherent to the “conventional” sensory feedback transmitted through the peripheral neural pathways. In essence, the EMG biofeedback can be regarded as a simple feedforward simulation of a linearized prosthesis. It provides the subject with an estimate (prediction) of the grasping force, which will be developed when the hand contacts the object. This allows the subject to adjust the current online command (reafference-based control) even before the force begins developing (control based on the online sensory feedback).

In our previous work [11], we demonstrated that the velocity of prosthesis closing can be used for a predictive control of grasping force. In the present study, the subjects had access to this information indirectly, since they had a clear view on the virtual gripper. Yet, the EMG biofeedback still improved the performance of force control. One more possibility would be to provide the closing velocity explicitly, using a visual bar (as for the EMG). However, implementing the predictive force control using EMG rather than velocity has several advantages. First, the feedback on velocity belongs to a classic scheme, in which the system state is transmitted to the user. Therefore, the system dynamics is still in the loop, i.e., the modulation of velocity is limited by the system responsiveness to user commands, including both mechanical (e.g., inertia) and computational (e.g., command processing and implementation) factors. On the other side, the modulation of EMG is virtually instantaneous. Second, feedback on velocity is meaningless after contact, since the velocity becomes zero. Therefore, it cannot be used to assist force steering. Thirdly, the EMG biofeedback can be implemented using standard prosthesis components, while to transmit the velocity one needs a velocity sensor (gyroscope) or a position sensor, where the latter has to provide a signal good enough to allow differentiation (which is not the case in Michelangelo Hand).

The aim of the current study was to describe the approach and test the concept feasibility. Therefore, the feedback was provided using an ideal interface (visual bar). The same approach could be implemented using electrotactile stimulation by transmitting the information about the magnitude of the control signal through a single-channel intensity and/or frequency and/or multi-channel spatial modulation. In the latter case, multiple stimulation electrodes can be used to implement an electrotactile equivalent of the visual bar, i.e., each electrode is associated to a signal range, and the current level of EMG is communicated by the currently active electrode within the array. Since the prosthesis is linearized, this also indicates the corresponding level of grasping force, once the prosthesis contacts the object. In order to produce a certain grasping force, the subject needs to activate the muscles so that a desired electrode starts stimulating. Providing the EMG biofeedback in this manner could result in a self-contained prosthetic system with an improved consistency of force control. The users would be able to produce a desired level of force repeatedly and reliably, eliminating the baseline variability as well as sudden large outliers that are characteristic for classic myocontrol [11]. Implementing the electrotactile EMG biofeedback to test these hypotheses is the work in progress.

This is not however a simple task since there a number of questions still to be answered. Ideally, two variables (EMG and force) need to be communicated to the user. This can be accomplished by using separate interfaces (dedicated electrodes) or the same interface with separate coding (see the video EMGBiofeedback.wmv and accompanying explanation in the Additional file 1). In any case, this adds an additional complexity to the system and also for the user, regarding his/her ability to perceive and utilize this information. In principle, however, the system can be simplified by implementing only the EMG biofeedback. Leaving out the force feedback would not affect the performance during routine grasping and the upward force steering, since in these cases the force corresponds to the level of EMG (linearized prosthesis). For the downwards force steering, the feedback would not communicate the current force level (force feedback), but the user would still be able to control the force transitions (EMG biofeedback). In any case, substituting the visual with a tactile interface, certainly decreases the quality of the information transfer. Pure spatial coding, for example, is intuitive for the subject to understand, but also limited to transmitting a set of discrete levels (each electrode one level). Mixed coding can increase the resolution but also the user cognitive effort. There are also limitations due to the technologies, such as, narrow dynamic range in electrostimulation due to discomfort at the higher stimulation intensities. All in all, it is still to be investigated how these factors (e.g., decrease in resolution, cognitive efforts) would affect the hereby demonstrated advantages of the EMG biofeedback as well as the overall user experience and acceptance of this approach.

Importantly, there are also limitations that must be considered when applying this approach in amputees. In the present study, the quality of myoelectric interfacing was improved by applying abrasive gel. In the real-life application, this is not available as only normal gel is used to moisturize the skin. Also, the quality of the myoelectric signals will depend on the condition of the residual limb (e.g., weaker muscles, scar tissue). This can compromise the myoelectric control in both cases, with classical force and EMG biofeedback. The impact of these factors and possible mitigation strategies have to be tested in the future work.

The consistency and accuracy of grasping reflect how reliable the system is in reproducing the user intention to grasp an object with a specific force, repeatedly and routinely. This is relevant for utility but also embodiment. Human hand is a reliable end effector, which responds promptly and consistently to user intentions, and if the artificial substitute would have similar characteristics, this would promote the effective substitution, both functionally and psychologically. In addition to improving the repeated grasping with the same force, the EMG biofeedback could also facilitate switching between forces across trials, as explained in the previous paragraphs. From the functional viewpoint, the provision of feedback makes the task demands explicit, i.e., the user can establish a mapping between daily life tasks and the grasping forces that are necessary to perform those tasks. If the user is also confident that he/she can generate those forces accurately and consistently, this could facilitate the optimal utilization of the prosthesis (economical grasping paradigm [20]). For example, if the EMG biofeedback is implemented using electrotactile stimulation with spatial coding at N levels, the user would know that he/she can generate N levels of force reliably. Through the use of the prosthesis, he/she would learn that specific tasks can be accomplished using certain forces, e.g., to pick grapes without squeezing them the force should be set at the level 2. Therefore, the user would determine the target force based on experience, and then generate that force fast and reliably using the EMG biofeedback interface.

The quality of force steering assessed through RMSE is relevant for object holding and manipulation. For example, when the force is gradually applied to a delicate object (e.g., wine glass) or when the force needs to be gradually decreased, e.g., for a smooth passing of an object from the prosthesis to a contralateral hand or to another person. In practice, unilateral amputees accomplish such sensitive tasks most often using a healthy hand, due to a poor controllability and other limitations [21]. A system that would improve the force modulation could increase the applicability of the prosthesis, and therefore improve the tradeoff between the efforts (training, mounting, maintenance) and gained functionality.

In the present experiment, some of the feedback cues that would normally be available to the prostheses users have been blocked. For example, most of the present day prostheses, including Michelangelo Hand, produce noise during movement and force modulation. However, it is unlikely that these additional feedback sources would affect the results and conclusions of the present study. Those cues indicate the prosthesis state (aperture and force), which was anyway clearly disclosed to the subjects using visual feedback (virtual gripper and force bar). Due to this and the phenomenon of visual dominance [22], it is unlikely that the additional cues, such as sound, would significantly improve the state assessment and therefore affect the overall performance. However, in a real-life application when the feedback is communicated through a practical electrotactile and/or vibrotactile interface and a visual assessment is non-ideal (e.g., viewing angle, occlusions), the incidental feedback could be more relevant. Importantly, this would mainly affect the force control using classic force feedback. From that point of view, the EMG biofeedback is rather robust, since the myoelectric command is adjusted based on the feedback about the state of the user (and not that of the prosthesis).

Myoelectric control can also be improved by applying specialized processing to the surface signals [23] and/or acquiring better signals through implanted interfaces [24]. Both approaches can substantially improve the stability and precision of the myoelectric waveforms. Importantly, these developments do not rule out the usefulness of the EMG biofeedback. More consistent signals lead to more consistent control, but the mapping between the subjective sense of muscle contraction and the resulting grasping force would still remain elusive. The latter connection can be made explicit by providing the EMG biofeedback to the user.

In this study, we have used a state of the art myoelectric hand, the latest model from Otto Bock. Importantly, the obtained insights and conclusions are general, since most myoelectric prostheses share the same principle of operation. Furthermore, the EMG biofeedback is not specific to force control. It could be utilized in a similar manner to facilitate the control of other prosthesis variables/degrees-of-freedom (e.g., velocity of opening/closing, velocity of wrist rotation).

## Conclusions

The present study proposes a novel paradigm to close the loop in a myoelectric prosthesis. In the classic approach, the feedback transmits to the user the state of the prosthesis (aperture, velocity and/or force), whereas in the novel method the feedback also informs the user about his/her own latent variables, i.e., the myoelectric signals he/she generates (EMG biofeedback). The experiments demonstrated that the provision of the EMG biofeedback improved the quality of force control both in routine grasping and force steering tasks, and both in able-bodied subjects and two amputees who were experienced users of myoelectric prostheses. With the EMG biofeedback displayed as a visual bar on the computer screen, the subjects could see and modulate the current level of their muscle activity, and thereby explicitly control the command they send to the prosthesis. In the conventional approach, the myoelectric signals are latent variables, which can be controlled only by using indirect cues, such as subjective experience (sensation of muscle contraction) and/or observable consequences (e.g., prosthesis movement). These sources are however unreliable, especially due to the inherent variability of the myoelectric signals recorded using surface electrodes. EMG biofeedback allows the user to improve the precision and accuracy of myoelectric commands using active control, i.e., fast local loop in which the user modulates the strength of muscle contraction based on the online EMG biofeedback. The present study demonstrated the feasibility, and the next step is the implementation of this approach using practical interfaces, such as electrotactile stimulation and augmented reality glasses, and the validation in a larger pool of subjects. Therefore, there are many practical questions still to address (e.g., functional gain vs. user efforts vs. acceptability), but the present results are very optimistic. The prosthesis equipped with the EMG biofeedback might increase the user confidence in the system, by allowing consistent and reliable force control, and this can improve the utility, embodiment and ultimately the acceptance rate. Furthermore, the EMG biofeedback could be also considered as a temporary add-on to the prosthesis, an instrument for training the subject to exploit the natural feedback from his/her own muscles for the closed-loop prosthesis control.

## Additional file

Additional file 1:
**The file contains a short movie (EMGBiofeedback.wmv) showing an amputee subject modulating the force of a prosthesis while holding an object.** The force feedback and EMG biofeedback were implemented using electrotactile stimulation. The movie is explained in more detail in the accompanying text file (EMGBiofeedback.doc).

## Abbreviations

EMG: Electromyography
MVC: Maximum voluntary contraction
IQR: Inter-quartile range
RMSE: Root mean square error
