# Supplementary material for: EMG Biofeedback for online predictive control of grasping force in a myoelectric prosthesis
Source: J Neuroeng Rehabil. 2015 Jun 19;12:55. doi: 10.1186/s12984-015-0047-z (PMC4485858; doi:10.1186/s12984-015-0047-z)
Supplement: Additional file 1: — The file contains a short movie (EMGBiofeedback.wmv) showing an amputee subject modulating the force of a prosthesis while holding an object. The force feedback and EMG biofeedback were implemented using electrotactile stimulation. The movie is explained in more detail in the accompanying text file (EMGBiofeedback.doc). [file 12984_2015_47_MOESM1_ESM.zip › EMGBiofeedback.docx]

A SHORT EXPLANATION FOR THE MOVIE EMGBiofeedback.wmv

The movie is an example of how some of the challenges (see Discussion) regarding the practical implementation of the EMG biofeedback can be addressed. The amputee subject used standard two-channel myoelectric control. To increase the force, the subject activated flexor muscle so that normalized myoelectric input surpassed current normalized grasping force (non-backdrivable prosthesis). To decrease the force, the subject activated extensor muscle above the threshold so that the hand started opening and releasing the grip. Due to the nature of the prosthesis operation, the grasping force increased/decreased through a set of discrete force levels.

To use a single interface for both force and EMG biofeedback, a mixed coding was implemented, i.e., spatial for force, and frequency and intensity modulation for EMG. The force was coded using six concentric electrodes, i.e., each electrode when active communicated to the subject that the grasping force was within the corresponding force range. The force ranges for the electrodes were selected to encompass the discrete levels generated by the prosthesis during increase/decrease (non-equidistant division of the full force range). The myoelectric activity was transmitted by modulating the frequency and intensity of stimulation at the currently active electrode. Therefore, the position of the currently active electrode within the array indicated to the subject the current level of grasping force, while the frequency and intensity of stimulation communicated the myoelectric input. As explained before, the most important information for the user when increasing the force is the difference between the momentary myoelectric input and the current force level. When this difference becomes greater than zero (myoelectric signal > current force), the prosthesis will increase the force. Therefore, the EMG biofeedback was implemented to communicate not the absolute value of the myoelectric input but its distance with respect to the current force. The higher was the muscle activation the higher the frequency, with the maximum frequency (100 Hz) indicating to the subject that the muscle activity reached the current level of grasping force. That is to say that the stimulation frequency range (from 10 to 100 Hz) was mapped linearly to the normalized distance i.e., 1 indicating subthreshold myoelectric activity and 0 signaling that the myoelectric input reached the current grasping force. To emphasize the important event (i.e., myoelectric command = grasping force), the stimulation intensity was also increased from low to high value when the normalized distance became lower than 0.1. The intensity values were determined beforehand for each electrode so that the elicited sensations were comfortable and easy to distinguish. When the subject felt that the stimulation is high and at maximum frequency (100 Hz), he knew that a further increase in the muscle activation would likely trigger the increase in the grasping force. Due to the prosthesis mechanism, this was not a completely deterministic process; rather, the stimulation indicated to the subject that the force could change at any moment during the further increase. A similar approach was implemented for the force decrease. As soon as the extensor activation crossed the threshold, the stimulation was delivered at the high intensity and frequency of 100 Hz to indicate to the subject that force decrease was imminent. Therefore, the EMG biofeedback informed the subject about the moment in which the prosthesis would start reacting to his command.

The laptop next to the prosthesis shows the visual representation of the electrical activity on each electrode (eight electrodes are depicted but six were used for the test). The first bar within each electrode represents stimulation intensity and the second frequency. The currently active electrode is the one with the non-zero intensity bar. The task for the subject was to close the hand and increase the force, step by step, through all six force levels in both directions. Importantly, the subject was not looking into the setup (laptop and hand). Note how at each level of force, the subject increases his muscle contraction gradually from the low level (muscle almost relaxed, frequency bar close to zero), attending to the frequency and intensity of stimulation, to carefully “push” the prosthesis over the reaction threshold (maximum frequency) and onto the next force level. The current grasping force assessed by the force sensor embedded into the prosthesis is shown as the yellow signal in the right panel. The y-axes is normalized from 0 to 100% of prosthesis force.
